# Proximate composition and sensory evaluation of gluten-free bar formulated from rice and soybean flour

**DOI:** 10.7717/peerj.21101

**Published:** 2026-04-14

**Authors:** Marwan El-Deyarbi, Mahmoud Abughoush, Shaimaa Hegazy, Abeer Aljaoune, Shorouk Aldeyarbi, Hamza Aljuboori, Huda Alnuaimi, Abdulla Almatrooshi, Zelal S. Adi, Ashraf A. Hussein, Alaa E. Nafea, Imranul H. Choudhury

**Affiliations:** 1 Department of Pharmacology & Therapeutics, College of Medicine and Health Sciences, United Arab Emirates University, Al Ain, United Arab Emirates; 2 Science of Nutrition and Dietetics Program, College of Pharmacy, Al Ain University, Al Ain, United Arab Emirates; 3 Faculty of Science, Port Said University Faculty of Science, Port Said, Egypt; 4 College of Medicine, Ajman University of Science & Technology, Ajman, United Arab Emirates; 5 Clinical Nutrition and Dietary, Abu Dhabi University, Abu Dhabi, United Arab Emirates; 6 Division of Endocrinology, Abu Dhabi University, Abu Dhabi, United Arab Emirates

**Keywords:** Gluten-free, Snack bar, Gluten intolerance, Celiac disease, Proximate, Sensory, Colorimetric analysis

## Abstract

**Background:**

The growing prevalence of celiac disease and gluten intolerance is driving up the need for gluten-free food choices suitable for both adults and children.

**Objective:**

This research evaluates the formulation of a gluten-free bar, utilizing rice flour, soybean flour, or a composite blend, with other enriching elements.

**Methods:**

Five different gluten-free bars were prepared from varying ratios of rice and soybean flour, and their proximate composition, colorimetric, and sensory properties were compared to wheat-based control bar.

**Results:**

The sensory analysis revealed that the formulation with 10% soybean and 30% rice blend ratio showed an overall higher acceptability score over the other formulations and compared to the control bar (total mean score = 45.72 and 42.94, respectively). Moreover, it has a favorable proximate composition of 10.5% protein, 4.6% fiber, and only 19.9% fat content compared to other formulations. The 40% rice flour formulation had the least sensory and chemical attributes compared to other formulations.

**Conclusions:**

The 10:30 soybean-rice formulation with fortifying ingredients achieved superior consumer acceptability while maintaining an optimal proximate profile, establishing an evidence-based benchmark for the industrial scale-up of high-protein, gluten-free bars. However, further research is required to optimize shelf-life stability for commercial distribution.

## Introduction

Hectic lifestyles and the growing demand for convenient, ready-to-eat healthy meals and snacks have encouraged the food manufacturing industry to produce foods like nutrition bars that combine vital nourishment with convenience. Cereal bars, emerging approximately a decade ago, continue to serve as a readily available and nutritionally beneficial alternative food source (Sharma et al., 2014). The inclusion of seeds, such as muskmelon and pumpkin, further enhances the nutritional profile of these bars by offering a beneficial blend of lipids, carbohydrates, and proteins that support energy levels and can even serve as meal replacements for individuals with busy schedules. Beyond their widespread availability in convenience stores, these bars are easily stored and suitable for everyday carrying in pockets and bags (Ahmed, Al-Jasas & Siddiq, 2014).

The global prevalence of celiac disease is increasing, affecting approximately 2.9% of the general population, with a higher frequency observed in females and individuals with co-existing autoimmune conditions (Gatti et al., 2024). This increasing burden necessitates diverse solutions aimed at reducing the health burden of this lifelong disorder. Many conventional snacks and energy bars contain gluten protein, found in wheat, barley, and rye, causing symptoms such as diarrhoea, bloating, gas, and general gastrointestinal distress, and often resulting in perpetual weight loss in individuals with celiac disease, compelling them to adhere to a lifelong gluten-free diet (Cabanillas, 2020). Moreover, recent studies have shown that a gluten-free diet can significantly benefit those with gluten-related illnesses by enhancing overall wellness, reducing chronic inflammation, and improving gastrointestinal health (Aljada, Zohni & El-Matary, 2021).

The availability of high-quality gluten-free nutritional products benefits not only celiac disease patients but also individuals with non-celiac gluten sensitivity, wheat allergies, diabetes or those adopting a gluten-free diet as a healthy lifestyle choice (El-Deyarbi et al., 2024; Pietzak & Kerner, 2012). In response to these dietary demands, the food industry has significantly increased its production of gluten-free products from gluten-free and allergen-free ingredients such as rice and soybean flour (Rai, Kaur & Chopra, 2018). Consequently, the estimated mean annual growth rate of gluten-free bars market is projected to increase by 7.2% from 2025 to 2030 (Gluten-Free Products Market Size, Trends & Forecast to 2029, 2024).

Available gluten-free products, including bread, biscuits, cookies, and other confectionery items, are often nutritionally imbalanced, deficient in essential vitamins and minerals, and higher-priced than their gluten-containing counterparts (Mehtab et al., 2024). To provide an affordable option while maintaining nutritional equilibrium, many studies have focused on developing various gluten-free food items, such as pasta, doughnuts, and cinnamon rolls, using lower cost ingredients with balanced nutrient profiles, specifically for celiac disease patients (AL-Othman et al., 2023; Maghaydah et al., 2024, 2022).

For the production of gluten-free products, gluten-free rice flour can be a valuable alternative, offering not only high digestible carbohydrate content and hypoallergenic properties but also other health advantages (Al Shehry, 2016). Furthermore, Wesley, André & Clerici (2021) reported that biscuits prepared with rice demonstrated high consumer acceptability and had a favorable impact on their sensory characteristics, making them suitable for celiac individuals. Soybean flour acts as valuable cereal substitute and can be an essential component of functional foods. Soybean proteins contain all essential amino acids, approximately four times that of wheat and six times that of rice grain, and serve as an excellent source of vitamins and minerals (Islam et al., 2007; Serrem, de Kock & Taylor, 2011) with a digestibility value of 91.41% (Zhao et al., 2014). Additionally, it can enhance product quality and shelf life (Ahmad et al., 2014; Mohamed et al., 2006).

Other ingredients have been added to the gluten-free bars to fulfill the nutritional needs of adults and children, such as eggs, yogurt, and honey. For instance, egg proteins provide the essential amino acids required by the human body. Incorporating yogurt significantly improves the bar’s nutritional profile by supplying calcium, probiotics, and anti-inflammatory compounds crucial for bone health and optimal gastrointestinal function. Moreover, its inclusion enhances the gluten-free bar’s structure, tenderness, and antioxidant activity (Graça et al., 2020). Honey, a natural sweetener, is a rich source of antioxidants, enhancing the final product’s palatability, sensory quality, shelf life, and nutritional value (Ajibola, Chamunorwa & Erlwanger, 2012; Gheldof & Engeseth, 2002).

While previous studies have explored non-wheat flour applications, for example tested maize and soybean flour blends for gluten-free biscuits (Akubor et al., 2023); mixed rice and soybean flour with starches for gluten-free breads (Taghdir et al., 2017), or focused on mixing different rice and bean products in biscuit formulation (Wesley, André & Clerici, 2021), research is limited, making a direct comparison of rice and soybean flours *vs* traditional wheat flour in a gluten-free food formulation challenging. Furthermore, few studies have integrated ingredients like yogurt (Graça, Raymundo & Sousa, 2020) and almond nuts (Stoin et al., 2018) to holistically improve the flavor, mouthfeel, and nutritional properties of these alternative gluten-free flour products.

This study aimed to formulate gluten-free bars from affordable ingredients such as rice flour and soybean flour in varying proportions, with defined amounts of other ingredients such as eggs, yogurt, almond nuts and honey and compared them with a control bar made from organic whole wheat flour (containing gluten) to develop a valuable alternative for gluten-sensitive individuals. The primary objective of the current study was to characterize each gluten-free bar formulation in terms of proximate composition and sensory properties, and the second objective was to determine the optimal formulation based on their proximate composition and sensory attributes.

## Materials and Methods

### Sample procurement

The formulation of the base ingredients added to the bar formulations was selected from the local market to ensure the availability of such ingredients in other similar markets. Fixed amounts of low-fat, unsweetened yoghurt from Almarai company, Kingdom of Saudi Arabia (calories = 0.5765 Kcal/g, total lipid = 0.0118 g/g, protein = 0.047 g/g, carbohydrate = 0.0647 g/g), egg yolk from Al Ain Farms, UAE, unsalted butter from Lurpak company, Danish (calories = 7.47 Kcal/g, total lipid = 0.82 g/g, protein = 0.006 g/g, carbohydrate = 0.007 g/g), and xanthan gum that were established by a preliminary study were used in the formulated bars.

Other ingredients such as analytical-grade polysaccharides (inulin) bought from Muscat Chemical Company (Oman), bicarbonate, Manuka honey from New Zealand (calories = 14.1 Kcal/g, total lipid = 0 g/g, protein = 0.003 g/g, carbohydrate = 0.82 g/g, UMF10+), salt (Nezo, Netherlands), vanilla extract, and chopped almond nuts from Bayara company, UAE (calories = 24.06 Kcal/g, total lipid = 0.49 g/g, protein = 0.21 g/g, carbohydrate = 0.2 g/g, fibers = 0.12 g/g) were added to the bar formulation.

### Preliminary base ingredients study

A preliminary study was conducted to determine the optimal quantity of the base ingredients for the main experiment. Wheat flour (containing gluten) and other base ingredients were prepared in different proportions as shown in Table 1. The composition of the most preferred sample was then selected for the gluten-free bar formulation.

**Table 1:** The amount of flour and base ingredients in the preliminary analysis.

Base ingredients	Control #1	Control #2	Control #3
Wheat flour–g	80	80	80
Low-fat yoghurt–g	15	30	45
Egg (yolk)–g	15	30	45
Butter–g	15	30	45
Xanthan gum–g	0.5	1	1.5
Inulin–g	5	5	5
Honey–g	10	10	10
Salt–g	1	1	1
Bicarbonate–g	0.5	0.5	0.5
Vanilla extract–g	0.5	0.5	0.5
Almond nuts–g	12	12	12

**Note:**

g, gram.

Three different formula concentrations have been prepared for the initial analysis based on the 1:2:3 percentage level (Table 1). For the initial analysis, a panel of ten trained staff members (seven faculty and three senior students) evaluated the three control samples using the same hedonic evaluation form used in the final sensory analysis to determine the most suitable formula that met the research objective.

These control samples contained low-fat yogurt, egg yolk, and butter in the amounts of 15, 30, and 45-g using the same preparation standard. The panelist consensus on control #2 considers it the best formula with a compatible taste and aroma, in addition to the appropriate firmness and mouthfeel. Furthermore, adding 1 g of xanthan gum to the formula enhanced the physical properties and was considered suitable in terms of the nutritional and chemical properties of the final product.

### Flour formulations’ mixtures

For the study, two organic gluten-free flours (rice, and soybean flours) and organic whole wheat flour were sourced from the local market (Phalada Pure & Sure Flour, India) (Table 2). Each flour had a valid expiration date and was stored at room temperature (between 23 °C and 27 °C) until it was mixed and used for bar cooking.

**Table 2:** Nutritional value of flours in the formulated bars.

Ingredients	Per 100 g of flour
Wheat flour (control)	
Calories	366 Kcal/g
Total lipid	2.1 g
Protein	14 g
Carbohydrate	74.2 g
Fibre	11.5 g
Sodium	17 mg
Estimated moisture content	9.68 g
Gluten-free rice flour	
Calories	372 Kcal/g
Total lipid	1.8 g
Protein	8.7 g
Carbohydrate	78.8 g
Sodium	1.9 mg
Estimated moisture content	10.7 g
Gluten-free soybean flour	
Calories	486 Kcal/g
Total lipid	23 g
Protein	36.2 g
Carbohydrate	36 g
Fibre	10.1 g
Sodium	12 mg
Estimated moisture content	4.79 g

**Note:**

Kcal/g, kilocalorie per gram; g, gram.

For this study, five different gluten-free bar formulations (F1–F5) were formulated using five different gluten-free flour mixtures from rice and soybean flour (based on 1:3, 1:1, and 1:0 ratios) in addition to the control bar from wheat flour only (Table 3).

**Table 3:** Proportions of flour formulations’ mixtures and the control bar.

Ingredients	Control	F1	F2	F3	F4	F5
Wheat flour–g (%)	80 (40%)	–	–	–	–	–
Rice flour–g (%)	–	20 (10%)	40 (20%)	60 (30%)	80 (40%)	–
Soybean flour–g (%)		60 (30%)	40 (20%)	20 (10%)	–	80 (40%)
Base ingredients^¥^–g (%)	120 (60%)	120 (60%)	120 (60%)	120 (60%)	120 (60%)	120 (60%)
Total–g	200 g	200 g	200 g	200 g	200 g	200 g

**Note:**

¥ Control #2 (Table 2); F, Formulation; g, gram.

### Gluten-free bar formulation

**Figure 1:**
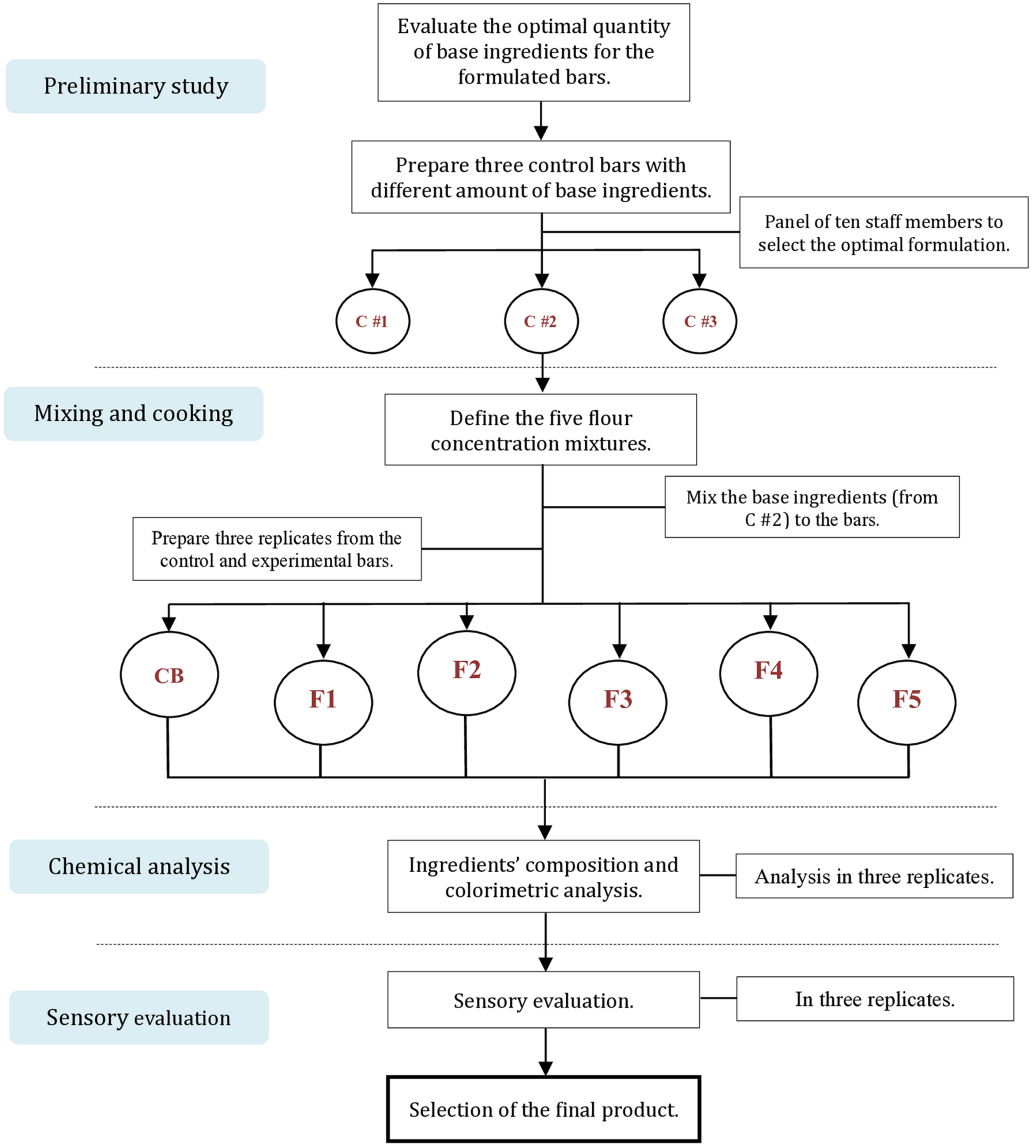
The methodological approach in the selection and analysis of the final gluten-free bar product. Control bars in preliminary analysis C #1: Control bar #1; C #2: Control bar #2; C #3: Control bar #3. Experimental bars are presented by CB: Control (100% wheat flour); F1 (25% rice, 75% soy); F2 (50% rice, 50% soy); F3 (75% rice, 25% soy); F4 (100% rice flour); and F5 (100% soy flour).

Five different formulations from fixed gluten-free rice and soybean flour concentrations, in addition to the control bar from organic wheat flour (containing gluten), were mixed with the base ingredients and baked. Additionally, three replicates of both the control and experimental bars were prepared using the same standard procedure and then baked for analysis in triplicate (Fig. 1).

The experimental bars and the control bar went through proximate analysis, followed by a qualitative approach evaluation by sensory evaluation to verify the quality of the final product in terms of aroma, taste, and overall consumer acceptance.

### Ingredients mixing and cooking

The ingredients (wheat and base ingredients) were weighed using a calibrated scale (Bel, Model M214Ai, Italy). All dry ingredients, including flour, salt, xanthan gum, inulin, and bicarbonate, were mixed in a 3.5 L stainless steel mixing bowl. Afterwards, the mixed ingredients were stirred in the pre-weighed yolk, vanilla, melted butter, and yoghurt until smooth and finally mixed with chopped almond nuts. The base ingredients were added to the six formulations based on the control #2 formula that was determined in the preliminary analysis (Table 3).

For cooking, the mixture was spread evenly in a six cavity (6.5 cm)^2^ carbon steel baking tray (18.7 cm × 26.5 cm) pretreated with butter. The tray was allowed to settle down for 10 min at room temperature of 27 °C before baking to form a dry, non-sticky external skin. In a preheated oven at 200 degrees Celsius, the mixture was baked for 10 min until the outer layer of the bar appeared dry, shiny, and distinctly crackled.

### Proximate analysis for bar composition and caloric value

Prior to analysis, formulated bars (Control [CB], F1, F2, F3, F4, and F5 bars) were first ground up in a mortar and pestle for examination. The proximate analysis included lipids, carbohydrate, crude fiber, protein, moisture and ash content analysis (Feldsine, Abeyta & Andrews, 2002; Thangaraj, 2016), and all analyses were conducted in three replicates for each bar to ensure accuracy.

Protein analysis was determined by the improved Kjeldahl method for indirect protein analysis using selenium catalyst, and to determine total protein content, the total nitrogen in each bar was multiplied by a traditional conversion factor of 6.25 (Aguirre, 2023). The percentage of crude lipid in grams was determined by the Soxhlet method using petroleum ether (40–60 °C) as the solvent and calculated by the below equation (Arunachalam, Saravanan & Parimelazhagan, 2011):

${{\rm Crude\; lipid} = \left[ {\left( {{\rm weight\; of\; empty\; flask\; } - {\rm the\; weight\; of\; flask\; and\; extracted\; fat}} \right)\\ /{\rm weight\; of\; sample}} \right]\; \times 100.}$

The crude fiber was calculated using the digestion method, digesting bar samples with dilute acid, followed by digestion with a diluted base. The percentage of moisture content was measured gravimetrically by drying the sample to a constant weight and was determined using the AOAC analytical method (Thiex, 2009). In addition, the Muffle furnace method was used to estimate the ash content by incinerating each sample at 450 °C for 3 h using a porcelain crucible until free from carbon.

After all, crude carbohydrate content was calculated by subtracting moisture, ash, total protein, lipids, and fiber contents from the total content. For the total caloric values in each bar, the Atwater general factor system was used to compute energy values for protein (4 kcal/g), lipid (9 kcal/g), and carbohydrate (4 kcal/g) (European Parliament and of the Council, 2011).

### Color analysis

Color analysis was carried out on the control and experimental bars’ crumb to determine the physical characteristics of the bars using a Minolta colorimeter (model CR-300, Tokyo, Japan). For analysis, the average value of three replications at different points in direct contact with the crumb region for each bar was calculated and recorded using the CIELL*a*b color method after calibration using the same standard on a white plate. The color scheme of L*a*b consists of one lightness component (L*) ranging from 0 to 100 (black to white, respectively) and two chromatic components: the (a*) component ranging from −a to +a (red to green, respectively) and the (b*) component ranging from −b to +b (blue to yellow, respectively) (Wesley, André & Clerici, 2021). Color analysis of the standard white plate was measured at two different points, in addition to the external color of both sides. The L*a*b values of the white standard plate were L = 98.2, a = +0.11, and b = +1.76. The whiteness index (WI) and the browning index (BI) for the bars were measured based on the below two equations (Sakin-Yilmazer et al., 2013; Tuncel et al., 2014):

${\rm WI} = 100 - \sqrt {{{\left( {100 - {{\rm L}^{\rm *}}} \right)}^2} + {{\rm a}^{{\rm *}2}} + {{\rm b}^{{\rm *}2}}}$

${\rm BI} = [100 \times \left( {{\rm x} - 0.31} \right)]/0.172$

And ${\rm x} = \left( {{\rm a} + 1.75{\rm \; }{{\rm L}^{\rm *}}} \right)/\left( {5.645{\rm \; }{{\rm L}^{\rm *}} + {{\rm a}^{\rm *}} - 0.3012{\rm \; }{{\rm b}^{\rm *}}} \right)$

Moreover, we quantified the ΔE of each sample based on the CIE L*a*b color parameters of the control sample and determined as $\triangle {\rm E = }\sqrt {{{{\rm (L}_1^{\rm *} - {\rm L}_2^{\rm *})}^2} + {{{\rm (a}_1^{\rm *} - {\rm a}_2^{\rm *})}^2} + {{{\rm (b}_1^{\rm *} - {\rm b}_2^{\rm *})}^2}}$, were CIE L*_1_, a*_1_, and b*_1_ are the values of the control bar, and CIE L*_2_, a*_2_, and b*_2_ correspond to the values of each sample bar.

### Sensory analysis

Quantitative sensory descriptive analysis using hedonic evaluation of the formulated and control bars was conducted in the Pharmacy College at Al Ain University in the United Arab Emirates (UAE) and approved by research ethics issues committee in Al Ain University. A total of fifty participants joined the sensory panel (10 faculty staff and 40 students); they were aged 23.4 ± 9.8 years, and 78% were female and 22% were male. All participants signed the informed consent and were trained to recognize each sensory attribute and to use intensity scales consistently. Each evaluator received in a randomized serving order almost one centimeter of each bar (one bar from the control and one from each experimental bar), coded according to the sensory evaluation standard procedure (Momanyi, Owino & Makokha, 2020) with three-digit random codes. Bars were cool down for a minimum of 2 h and were served at room temperature of 27 °C in a single session (around 30 min) in the food science laboratory in the Pharmacy College of Al Ain University. Participants were asked to evaluate each bar sample for the following five quality features: (1) appearance, (2) aroma, (3) mouthfeel, (4) taste, and (5) overall acceptability, using a 9-point hedonic scale ranging from 1 = dislike extremely to 9 = like extremely (Meilgaard, Carr & Civille, 1999), and between each sample evaluation, they were instructed to drink mineral water to clear their palate and minimize the carryover effect from the previous sample.

### Statistical analysis

To fulfill normality and homogeneity of variance assumptions, we used complete randomized design (CRD) during proximate analysis (Quintana-Obregón et al., 2019) and randomized complete block design (RCBD) during the general acceptability testing (Akinwale et al., 2021). Statistical analysis was performed using SPSS software (version 26). One-way analysis of variance (ANOVA) was used to test for differences in sample means, and Tukey’s Honestly Significant Difference (HSD) was used to determine the significant differences among group means in the proximate, color, and sensory analysis. Differences are considered significant at a *p*-value of <0.05.

## Results

### Proximate composition

Proximate composition of the six formulated bars, including the control bar (CB, F1, F2, F3, F4, and F5), are presented in Table 4. A trend towards an overall increase in nutrition calories (Kcal/100 g) was observed in the formulated bars (F1, F2, and F3) as the proportion of rice flour increased when mixed with soybean flour (mean (±SD) 327.47 ± 29.57, 333.83 ± 34.68, and 338.21 ± 25.42, respectively). The highest caloric content was found in F4 (solely from rice flour), with a mean (±SD) of 339.67 ± 28.46; however, the differences from the control bar were not significant.

**Table 4:** Proximate analysis of bar samples reported (in g/100 g) in dry weight basis, except moisture.

Formulation	CB	F1	F2	F3	F4	F5
Kcal/100 g*	328.95 ± 25.73^a^	327.47 ± 29.57^a^	333.83 ± 34.68^a^	338.21 ± 25.42^a^	339.67 ± 28.46^a^	329.43 ± 31.84^a^
Lipid*	17.81 ± 1.56^d^	22.76 ± 2.05^ab^	21.37 ± 2.22^bc^	19.92 ± 1.49^cd^	17.97 ± 1.53^d^	24.47 ± 2.37^a^
Fiber*	3.60 ± 0.31^e^	6.79 ± 0.61^b^	5.69 ± 0.59^c^	4.55 ± 0.34^d^	3.31 ± 0.28^e^	7.98 ± 0.77^a^
Protein*	8.43 ± 0.74^e^	17.75 ± 1.59^b^	14.17 ± 1.46^c^	10.52 ± 0.79^d^	6.62 ± 0.57^f^	21.53 ± 2.08^a^
Moisture*	26.34 ± 2.31^a^	25.19 ± 2.26^a^	25.97 ± 2.69^a^	26.76 ± 2.01^a^	26.84 ± 2.29^a^	24.75 ± 2.39^a^
Ash*	0.99 ± 0.09^e^	2.39 ± 0.21^b^	1.191 ± 0.19^c^	1.143 ± 0.11^d^	0.91 ± 0.08^e^	2.89 ± 0.78^a^
Carbohydrate*	42.82 ± 5.01^a^	25.11 ± 6.72c	30.9 ± 7.17^bc^	36.81 ± 4.75^b^	44.34 ± 4.76^a^	18.37 ± 7.89^d^

**Notes:**

* Data is the average mean of three replicates ± standard deviation.

a–f Values of different columns with different superscript letters indicate significant differences (*p* ≤ 0.05) among values according to the Tukey method. Treatment formulations are presented by CB: Control (40% wheat flour); F1 (10% rice, 30% soybean); F2 (20% rice, 20% soybean); F3 (30% rice, 10% soybean); F4 (40% rice flour); and F5 (40% soybean flour).

#### Lipid and carbohydrate content

Formulations F5 (40% soybean flour only) and F1 (30% soybean flour) revealed higher lipid concentrations (24.47% and 22.76%, respectively) but lower carbohydrate content (18.37% and 25.11%, respectively) compared to the control bar (CB: 17.8% lipid and 42.82% carbohydrate). Conversely, formulation F4 (40% rice flour only) showed the highest carbohydrate content and the lowest lipid content among the mixed formulations, with statistically significant results.

Additionally, there were significant differences (*p* < 0.05) in the percentage of carbohydrates across most of the gluten-free bar formulations and the control bar. The carbohydrate content of the study’s formulations was 25.11%, 30.9%, 36.81%, 44.34%, and 18.37% for F1, F2, F3, F4, and F5, respectively, compared to 42.82% for the control bar.

#### Fiber and protein content

Comparable results were shown in the formulations made from rice and soybean flour (F1, F2, and F3) with a decrease in the fiber content (6.79%, 5.69%, and 4.55%, respectively), as well as the protein content (17.75%, 14.17%, and 10.52%, respectively), with the decrease in the soybean flour content, and the results were significant compared to the control bar from wheat flour only (3.6% and 8.43%, respectively).

#### Moisture and ash content

The moisture content did not vary significantly among the different formulated bars, ranging from 25.19% (F1) to 26.8% (F4). However, formulation F4, from rice flour only, showed the highest moisture content compared to other bars (Table 4). Furthermore, the ash content in the formulated bars ranged from 0.91% to 2.89%. Formulation F5 (from soybean flour only) showed the highest ash content among all formulated bars and the control bar, with statistically significant results.

### Color analysis

Table 5 presents the color parameters of the gluten-free bar formulations and the control. For the L* parameter (lightness), formulation F4 (made solely from rice flour) showed a significantly higher value compared to other bar formulations (57.18). Moreover, it was observed that the L* value decreased with an increasing proportion of soybean flour (and decreasing the rice flour proportion) in F1, F2, and F3 (33.86, 34.24, and 36.67, respectively). Regarding the a* parameter (redness), values ranged between 9.23 and 17.99, indicating an increase in bar redness with a higher proportion of soybean flour. Similarly, for the b* parameter (yellowness), the bar formulations exhibited values between 21.43 and 33.25, also signifying a greater yellowing effect as the soybean flour proportion increased.

**Table 5:** Color parameters using the CIE Lab system for gluten-free formulations and the control.

Formulation	CB	F1	F2	F3	F4	F5
L*	44.7 ± 0.12^b^	33.86 ± 0.35^de^	34.24 ± 0.38^d^	36.67 ± 0.40^c^	57.18 ± 0.39^a^	33.17 ± 0.45^e^
a*	12.3 ± 0.32^e^	16.4 ± 0.05^b^	15.95 ± 0.08^c^	15.13 ± 0.04^d^	9.23 ± 0.33^f^	17.99 ± 0.06^a^
B*	21.43 ± 0.41^d^	32.85 ± 0.18^ab^	32.14 ± 0.10^b^	31.72 ± 0.22^bc^	29.79 ± 0.44^c^	33.25 ± 0.36^a^
WI*	39.43^b^	24.35^d^	25.09^d^	27.57^c^	47.03^a^	23.22^d^
BI*	264.31^d^	407.98^ab^	395.13^b^	366.67^c^	263.53^d^	423.81^a^
ΔE	–	16.27^b^	15.41^c^	13.36^d^	15.33^c^	17.47^a^

**Notes:**

* Data is expressed in dimensionless units, and it is the average mean of three replicates ± standard deviation.

a–e Means within the same row with different superscripts are significantly different (*p* < 0.05) according to the Tukey method. Treatment formulations are presented by CB, Control (40% wheat flour); F1 (10% rice, 30% soybean); F2 (20% rice, 20% soybean); F3 (30% rice, 10% soybean); F4 (40% rice flour); and F5 (40% soybean flour); L, lightness; a, redness; B, yellowness; WI, whiteness index; BI, browning index; ΔE, Delta E.

The Whiteness Index (WI) of formulation F4 (made solely from rice flour) was significantly higher than the other formulations (47.03). Conversely, the F5 bar (made from soybean flour) showed lower whiteness (23.22) compared to other formulations and the control bar. For the browning index (BI), an inverse relationship to WI values was observed in formulations F1, F2, and F3 (407.98, 395.13, and 366.67, respectively), and the F5 bar showed the highest value (423.81), while the control bar had the lowest (264.31). Regarding Delta E (ΔE) values, formulation F3 showed the lowest value (13.36), followed by F4 (15.33) and F2 (15.41). Generally, F4 was the formulation with the highest luminosity (lightest color), whereas F5 obtained the lowest value of coloration (darkest color) (ΔE = 15.33 and 17.47, respectively) (Table 5).

### Sensory analysis

**Figure 2:**
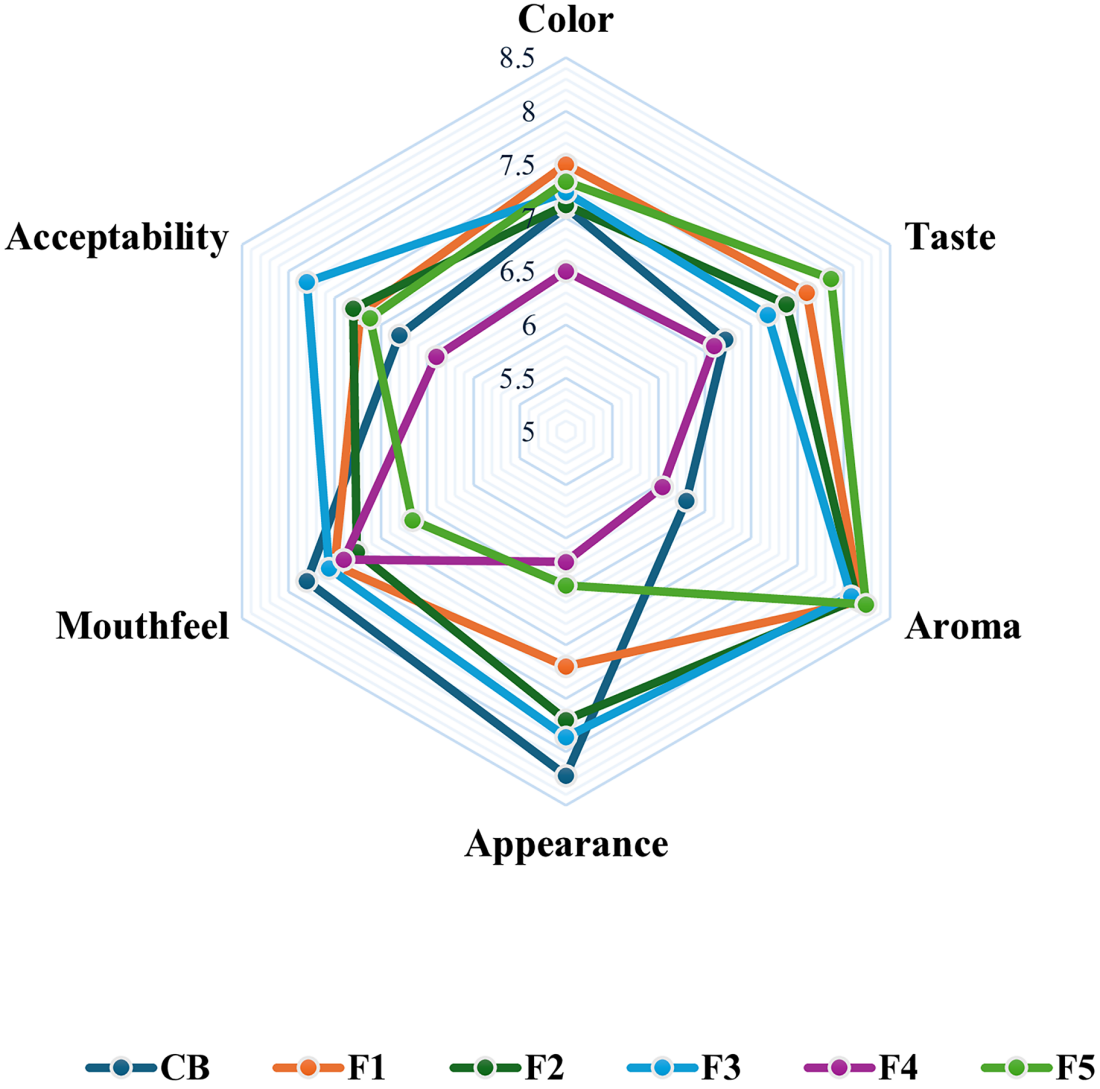
Formulated bars acceptability using a nine-point hedonic scale. Treatment formulations are presented by CB, Control (40% wheat flour); F1 (10% rice, 30% soybean); F2 (20% rice, 20% soybean); F3 (30% rice, 10% soybean); F4 (40% rice flour); and F5 (40% soybean flour).

Following manufacturing, bars were allowed to cool for at least 2 h and were introduced to fifty evaluators at room temperature (27 °C) for immediate sensory evaluation (Fig. 2).

Figure 2 presents the overall analysis of the gluten-free bars’ sensory characteristics. The total scores from these evaluators revealed that formulation F3 was higher in both sensory and physical properties compared to other formulations, achieving a total mean score of 45.72, and outperformed the control bar (CB), which received a total score of 42.94. Comparable results were obtained for overall acceptance, with F3 scoring the highest (7.8) and F4 the lowest (6.4). Moreover, the sensory analysis results showed that the highest formulation in the mean appearance score was for the control bar (8.22); however, formulations with rice wheat proportion more than 50% (F2 and F3) achieved a higher score (7.7.7 and 7.86, respectively) compared to other formulated bars, where the lowest score was for F4 (6.22), which was made from rice flour only. The high level of sensory evaluation of F3 in comparison to other formulations in the study and control bar was illustrated in Fig. 2.

## Discussion

### Proximate composition

In the investigated bars, the chemical analysis revealed that the final products varied in their lipid, protein, and fiber content relative to the dietary reference values (European Food Safety Authority (EFSA), 2017). This variation was directly attributable to the differing concentrations of wheat, rice, and soybean flours used in the formulations. Regarding the nutrition calories (Kcal), the European and other national authorities recommend approximately 300 calories, 8 to 10 g of protein (25–50% of total energy), and 100% of the recommended daily allowance for at least 12 important vitamins and minerals should be included in each serving of meal replacement products (Cheftel, 2005). The high Kcal content found in the formulated bars fulfils the requirements for high-energy bar classification, and may be linked to the high Kcal and nutritional value of the added ingredients such as butter, egg yolk, and honey in these formulations (McKillop et al., 2021). In this study, the Kcal content per 100 g of the manufactured bars ranged from 339.67 (F4) to 338.21 (F3). The increased energy content in the rice-based bars is primarily driven by the carbohydrate-rich profile of the rice flour. This trend aligns with Jan et al. (2021), reflecting the inherent carbohydrate density of rice flour, which provides a more significant caloric contribution than the protein-rich soybean (F5) or standard wheat (control) bars.

The soybean flour, from ground soybeans, has an exceptionally high-fat content compared to other known flours (Messina, 1997), and contains up to 38% protein (Bansal & Kapoor, 2015) indicating the higher nutritional value and energy content. This explains the higher lipid content in F5 and F1 formulations (24.47% and 22.76%, respectively) compared to other formulations, including the control bar. Similar results were reported by Taghdir et al. (2017) in gluten-free bread made from soybean flour. Moreover, in the three formulations’ mixtures of rice and soybean flour, F1 bars reported a higher content of protein compared to F2 and F3 bars, which was attributable to the difference between the soybean flour added in each formulation as well as in the study carried out by Bansal & Kapoor (2015) for bread made with different soybean flour blends. Signifying that soybean flour may act as an effective fortifying agent that improves nourishment without compromising the product’s acceptability when used at appropriate substitution levels. As expected, the highest protein content was found in the F5 formulation (21.53%), made from soybean flour only.

Moreover, out of all the formulations, the F4 formulation from rice (40%) revealed the highest carbohydrate and the lowest protein contents (44.34% and 6.62%, respectively) compared to other formulations containing soybean or wheat flour. This result may be attributed to the high starch content (carbohydrates) and low protein content in formulations made solely from rice flour, which may produce protein-energy malnutrition, especially in children (Mugalavai, Aduol & Onkware, 2021).

Fiber plays a crucial role in preventing obesity, diabetes, and cardiovascular disease, which are usually less in gluten-free products (El-Deyarbi et al., 2025; He et al., 2022; Ludwig et al., 1999; Wesley, André & Clerici, 2021). Analysis outcomes from mixed gluten-free bar formulations (F1, F2, and F3), presented in Table 4, showed values ranged from 4.55 to 6.79%, with significant difference from the control bar from wheat flour. These results were superior to values reported in other studies (Ayo et al., 2014; Farzana & Mohajan, 2015; Taghdir et al., 2017), because of the higher proportion of soybean flour in this study, indicating that bars made from soybean and rice flour can provide the essential nutrients recommended for daily consumption in addition to being high in fiber. This ensures a balanced amino acid profile while promoting improved digestion and a healthy gut microbiome through high dietary fiber content.

In the presented results, the highest ash content was reported in the F5 formulation (2.89%) made from soybean flour, and the lowest in the F4 formulation (0.91%) made solely from rice flour, compared to the CB formulation (0.99%) from wheat flour, which was comparable to the results presented in similar studies (Maghaydah et al., 2024; Mahmoud, Mohdaly & Elneairy, 2015; Taghdir et al., 2017). However, the ash content in the mixed soybean and rice flour ranged from 1.14% in F3 to 2.39% in F1, considering that the F1 formulation is made from 75% soybean flour, with a similar trend found in other studies with increasing the soybean proportion (Taghdir et al., 2017). The elevated ash content reflects the significant mineral contribution of the soybean flour, resulting in a nutrient-dense bar that supports bone health, immune function, and energy metabolism.

### Color analysis

The results of the chromatic parameters, which are an important property for the acceptance of the product by consumers, are shown in Table 5. The wheat flour used in the study is white in color, which explains the high L* as the bar leans towards a lighter tone with some yellowness (b*) (44.7 and 21.43, respectively). Moreover, xanthan gum, which is incorporated into the baked bar to create a more cohesive and less crumbly bar, is also a white powder which aids to increase L* values and make the bar brighter. However, adding honey during baking significantly contributed to the browning of the bar due to Maillard reactions and caramelization (Ronie et al., 2023), and decreased L* values. In addition, adding inulin fiber, which is usually a white powder, also affects the bar’s water activity and baking properties and aids in the browning of the bar, while the fat and protein content of the added almond nuts decreases the L* value more.

While the Control Bar (CB) showed a high WI due to the wheat flour, there was a progressive increase in WI from F1 to F4. This rising trend is explained by the increasing proportion of white rice flour, which contributed to the brightness of the formulations. Conversely, a progressive decline in BI was observed from F1 through F4, with F1 exhibiting the highest value; this trend is attributed to the incremental addition of darker soybean flour, as well as the occurrence of Maillard reactions and caramelization during processing (Ronie et al., 2023). Moreover, the higher L* values and lower B* as well as a* values in the rice bars (57.18) were expected due to the natural white color of the rice flour and were similar to those found by Wesley, André & Clerici (2021) for biscuits made from gluten-free rice. On the other hand, the lower L* values and higher B* values in soybean bars (F5, F1, F2, and F3) (33.17) were due to the high protein content of the soybean flour and the contributed Maillard browning reactions (Ronie et al., 2023). Beside the light yellow to creamy white color of the soybean wheat contributed more to lower L* values.

### Sensory analysis

Food quality is a multidimensional concept encompassing objective factors, including hidden attributes such as nutritional value crucial for health and well-being, and subjective enjoyment of the product’s sensory properties, including appearance, aroma, taste, and mouthfeelor mouthfeel (Forde & de Graaf, 2022). The sensory analysis in this study showed that including soybean flour in gluten-free formulations has improved the aroma, and mouthfeel of the formulated bars compared to the control formulation (CB) with wheat flour and the F4 formulation from rice flour only. Moreover, regarding overall acceptance, the analysis findings showed that the 30 percent rice flour with 10 percent soybean flour (F3) was the most suitable and acceptable compound mixture regarding color (6.5), aroma (6.04), and mouthfeel (7.4), with similar results shown in other studies (Małecki et al., 2020). The success of T3 in most sensory analyses and overall acceptance (total mean score 39.16) can be due to the perfect fusion of the mild, comforting taste of rice with the nutty aroma of soybean, a blend that increased the final product’s aroma experience, mouthfeel, appearance, and overall acceptance.

The demand for gluten-free diets is expanding, driven by the increasing prevalence of celiac disease and gluten sensitivity. This research presents innovative formulations designed to enhance the proximate composition, sensory parameters, and overall quality of gluten-free bars made from soybean and rice flour. However, a limitation of this study was the unavailability of similar commercial gluten-free bars, which prevented us from highlighting the unique advantages of our developed bars against a benchmark product. Another limitation is the need for additional tests, particularly shelf-life studies, to evaluate the stability of the bars over time. This is crucial for assessing changes in aroma, mouthfeel, and microbial safety, especially considering the blend of additives added to the bars. Nevertheless, the study’s findings hold significant potential to influence the commercial production of gluten-free energy bars and offer novel suggestions for enhancing consumer appeal.

## Conclusion

This study showed that formulations from soybean and rice flours can significantly enhance proximate composition, colorimetric properties, and sensory attributes of gluten-free bars. Among the different formulations, formulation 3 (F3), with a 10% soybean and 30% rice blend ratio, emerged as the optimal treatment, achieving the highest scores for aroma and consumer acceptability. This study advances gluten-free food science by developing innovative soybean and rice flour blends designed to addresses the sensory limitations and nutrient gaps common in gluten-free bars. Moreover, by utilizing sustainable, cost-effective local crops, this research provides a scalable framework for producing a nutrient-dense, calorie-dense snack fortified with elevated levels of protein and dietary fiber tailored to the dietary needs of celiac patients or individuals with high physical activity levels while supporting the global transition toward plant-based food systems.

Future research should explore the incorporation of defatted soybean flour as a strategy to minimize lipid concentration and optimize the protein-to-energy ratio. This substitution could further concentrate the fiber and ash content while simultaneously enhancing oxidative stability, thereby extending the bar’s shelf-life.

## Supplemental Information

10.7717/peerj.21101/supp-1 Supplemental Information 1 Study Raw Data.

## Additional Information and Declarations

### Data Availability

The following information was supplied regarding data availability:

The raw data is available in the Supplemental File and Zenodo:

- El-Deyarbi, M. (2025). Formulation and Physicochemical Characterization of Gluten Free Snack Bars Utilizing Rice and Soy Flour. [Data set]. Zenodo. https://doi.org/10.5281/zenodo.17618363.

## References

[ref-1] Aguirre J (2023). The Kjeldahl method. The Kjeldahl Method: 140 Years.

[ref-2] Ahmad A, Hayat I, Arif S, Masud T, Khalid N, Ahmed A (2014). Mechanisms involved in the therapeutic effects of soybean (*Glycine Max*). International Journal of Food Properties.

[ref-3] Ahmed J, Al-Jasas FM, Siddiq M (2014). Date fruit composition and nutrition. Dates: Postharvest Science, Processing Technology and Health Benefits.

[ref-4] Ajibola A, Chamunorwa JP, Erlwanger KH (2012). Nutraceutical values of natural honey and its contribution to human health and wealth. Nutrition & Metabolism.

[ref-5] Akinwale RO, Odunlami LK, Eze CE, Oladejo AS (2021). Effectiveness of different alpha lattice designs in the evaluation of maize (*Zea mays* L.) genotypes in a rainforest agro-ecology. Heliyon.

[ref-6] Akubor PI, Onogwu OC, Okereke GO, Damak AMA (2023). Production and quality evaluation of gluten free biscuits from maize and soybean flour blends. European Journal of Nutrition & Food Safety.

[ref-7] Al Shehry GA (2016). Use of corn and quinoa flour to produce bakery products for celiac disease. Advances in Environmental Biology.

[ref-8] AL-Othman H, Maghaydah S, Abughoush M, Olaimat AN, Al-Holy MA, Ajo R, Al Khalaileh NI, Choudhury IH, Angor M (2023). Correction: AL-Othman et al. development and characterization of nutritious gluten-free doughnuts with lupin and inulin flours. Foods 2022, 11, 3237. Foods.

[ref-9] Aljada B, Zohni A, El-Matary W (2021). The gluten-free diet for celiac disease and beyond. Nutrients.

[ref-10] Arunachalam K, Saravanan S, Parimelazhagan T (2011). Nutritional analysis and antioxidant activity of palmyrah (*Borassus flabellifer* l.) seed embryo for potential use as food source. Food Science and Biotechnology.

[ref-11] Ayo JA, Ayo VA, Popoola C, Omosebi M, Joseph L (2014). Production and evaluation of malted soybean-acha composite flour bread and biscuit. African Journal of Food Science and Technology.

[ref-12] Bansal R, Kapoor K (2015). Physiochemical analysis of bread fortified with different levels of soya flour blends. International Journal of Pure & Applied Bioscience.

[ref-13] Cabanillas B (2020). Gluten-related disorders: celiac disease, wheat allergy, and nonceliac gluten sensitivity. Critical Reviews in Food Science and Nutrition.

[ref-14] Cheftel JC (2005). Food and nutrition labelling in the European union. Food Chemistry.

[ref-15] El-Deyarbi M, Ahmed L, King J, Adi ZS, Al Juboori A, Mansour NA, Al Nuaimi H, Beiram R, Aburuz S (2025). Impact of multifactorial interventions with medication and lifestyle optimization on patients with type 2 diabetes: a randomised controlled trial. PLOS ONE.

[ref-16] El-Deyarbi M, Ahmed LA, King J, Al Nuaimi H, Al Juboori A, Mansour NA, Jarab AS, Abdel-Qader DH, Aburuz S (2024). Effect of structured diet with exercise education on anthropometry and lifestyle modification in patients with type 2 diabetes: a 12-month randomized clinical trial. Diabetes Research and Clinical Practice.

[ref-17] European Food Safety Authority (EFSA) (2017). Dietary reference values for nutrients summary report.

[ref-18] European Parliament and of the Council (2011). Regulation (EU) no 1169/2011 of the European parliament and of the council of 25 October 2011 on the provision of food information to consumers, amending regulations (EC) no 1924/2006 and (EC) no 1925/2006 of the European parliament and of the council, and repealing commission directive 87/250/EEC, council directive 90/496/EEC, commission directive 1999/10/EC, directive 2000/13/EC of the European parliament and of the council, commission directives 2002/67/EC and 2008/5/EC and commission regulation (EC) no 608/2004. Official Journal of the European Union.

[ref-19] Farzana T, Mohajan S (2015). Effect of incorporation of soy flour to wheat flour on nutritional and sensory quality of biscuits fortified with mushroom. Food Science & Nutrition.

[ref-20] Feldsine P, Abeyta C, Andrews WH (2002). AOAC international methods committee guidelines for validation of qualitative and quantitative food microbiological official methods of analysis. Journal of AOAC International.

[ref-21] Forde CG, de Graaf K (2022). Influence of sensory properties in moderating eating behaviors and food intake. Frontiers in Nutrition.

[ref-22] Gatti S, Rubio-Tapia A, Makharia G, Catassi C (2024). Patient and community health global burden in a world with more celiac disease. Gastroenterology.

[ref-23] Gheldof N, Engeseth NJ (2002). Antioxidant capacity of honeys from various floral sources based on the determination of oxygen radical absorbance capacity and inhibition of in vitro lipoprotein oxidation in human serum samples. Journal of Agricultural and Food Chemistry.

[ref-24] Gluten-Free Products Market Size, Trends & Forecast to 2029 (2024). FB 2585. Markets and markets. https://www.marketsandmarkets.com/Market-Reports/gluten-free-products-market-738.html.

[ref-25] Graça C, Mota J, Lima A, Boavida Ferreira R, Raymundo A, Sousa I (2020). Glycemic response and bioactive properties of gluten-free bread with yoghurt or curd-cheese addition. Foods.

[ref-26] Graça C, Raymundo A, Sousa I (2020). Yogurt as an alternative ingredient to improve the functional and nutritional properties of gluten-free breads. Foods.

[ref-27] He Y, Wang B, Wen L, Wang F, Yu H, Chen D, Su X, Zhang C (2022). Effects of dietary fiber on human health. Food Science and Human Wellness.

[ref-28] Islam AFMT, Chowdhury MGF, Islam MN, Islam MS (2007). Standardization of bread preparation from soy flour. http://ggfjournals.com/assets/uploads/4.15-20_.pdf.

[ref-29] Jan N, Naik HR, Gani G, Bashir O, Hussain SZ, Rather AH, Zargar IA, Wani SM, Amin T (2021). Optimization of process for the development of rice flour incorporated low-gluten wheat based pretzels: evaluation of its physicochemical, thermal and textural characteristics. Journal of the Saudi Society of Agricultural Sciences.

[ref-30] Ludwig DS, Pereira MA, Kroenke CH, Hilner JE, Van Horn L, Slattery ML, Jacobs DR (1999). Dietary fiber, weight gain, and cardiovascular disease risk factors in young adults. The Journal of the American Medical Association.

[ref-31] Maghaydah S, Abu-Ghoush M, Hayajneh W, Iqbal S (2024). Development and characterization of high-fiber, gluten-free pasta for celiac disease patients. Applied Sciences.

[ref-32] Maghaydah S, Alkahlout A, Abughoush M, Al Khalaileh NI, Olaimat AN, Al-Holy MA (2022). Novel gluten-free cinnamon rolls by substituting wheat flour with resistant starch, lupine and flaxseed flour. Foods.

[ref-33] Mahmoud AA, Mohdaly AAA, Elneairy NAA (2015). Wheat germ: an overview on nutritional value, antioxidant potential and antibacterial characteristics. Food and Nutrition Sciences.

[ref-34] Małecki J, Tomasevic I, Djekic I, Sołowiej BG (2020). The effect of protein source on the physicochemical, nutritional properties and microstructure of high-protein bars intended for physically active people. Foods.

[ref-35] McKillop K, Harnly J, Pehrsson P, Fukagawa N, Finley J (2021). FoodData central, USDA’s updated approach to food composition data systems. Current Developments in Nutrition.

[ref-36] Mehtab W, Agarwal S, Agarwal H, Ahmed A, Agarwal A, Prasad S, Chauhan A, Bhola A, Singh N, Ahuja V (2024). Gluten-free foods are expensive and nutritionally imbalanced than their gluten-containing counterparts. Indian Journal of Gastroenterology.

[ref-37] Meilgaard MC, Carr BT, Civille GV (1999). Sensory evaluation techniques.

[ref-38] Messina MJ (1997). Soyfoods: their role in disease prevention and treatment. Soybeans.

[ref-39] Mohamed AA, Rayas-Duarte P, Shogren RL, Sessa DJ (2006). Low carbohydrates bread: formulation, processing and sensory quality. Food Chemistry.

[ref-40] Momanyi D, Owino W, Makokha A (2020). Formulation, nutritional and sensory evaluation of baobab based ready-to-eat sorghum and cowpea blend snack bars. Scientific African.

[ref-41] Mugalavai VK, Aduol KO, Onkware AO (2021). Nutritional characteristics of rice (*Oryza sativa* L.) composite flours obtained by food fortification. http://41.89.164.27:8080/xmlui/handle/123456789/1629.

[ref-43] Pietzak M, Kerner JA (2012). Celiac disease, wheat allergy, and gluten sensitivity: when gluten free is not a fad. Journal of Parenteral and Enteral Nutrition.

[ref-44] Quintana-Obregón E, San Martín-Hernández C, Muy-Rangel M, Vargas-Ortiz M (2019). Evaluation of mango peel powder (*Mangifera indica* L.) as an alternative for the generation of functional foods. TIP Specialized Journal in Chemical-Biological Sciences.

[ref-45] Rai S, Kaur A, Chopra CS (2018). Gluten-free products for celiac susceptible people. Frontiers in Nutrition.

[ref-46] Ronie ME, Mamat H, Abdul Aziz AH, Zainol MK (2023). Proximate compositions, texture, and sensory profiles of gluten-free bario rice bread supplemented with potato starch. Foods.

[ref-47] Sakin-Yilmazer M, Kemerli T, Isleroglu H, Ozdestan O, Guven G, Uren A, Kaymak-Ertekin F (2013). Baking kinetics of muffins in convection and steam assisted hybrid ovens (baking kinetics of muffin…). Journal of Food Engineering.

[ref-48] Serrem CA, de Kock HL, Taylor JRN (2011). Nutritional quality, sensory quality and consumer acceptability of sorghum and bread wheat biscuits fortified with defatted soy flour. International Journal of Food Science and Technology.

[ref-49] Sharma C, Kaur A, Aggarwal P, Singh B (2014). Cereal bars—a healthful choice a review. Carpathian Journal of Food Science & Technology.

[ref-50] Stoin D, Jianu C, Mişcă C, Bujancă G, Rădulescu L (2018). Effect of almond flour on nutritional, sensory and bakery characteristics of gluten-free muffins.

[ref-42] Thangaraj P (2016). Proximate composition analysis. Pharmacological Assays of Plant-Based Natural Products. Progress in Drug Research.

[ref-51] Taghdir M, Mazloomi SM, Honar N, Sepandi M, Ashourpour M, Salehi M (2017). Effect of soy flour on nutritional, physicochemical, and sensory characteristics of gluten-free bread. Food Science & Nutrition.

[ref-52] Thiex N (2009). Evaluation of analytical methods for the determination of moisture, crude protein, crude fat, and crude fiber in distillers dried grains with solubles. Journal of AOAC International.

[ref-53] Tuncel NB, Yılmaz N, Kocabıyık H, Uygur A (2014). The effect of infrared stabilized rice bran substitution on B vitamins, minerals and phytic acid content of pan breads: part II. Journal of Cereal Science.

[ref-54] Wesley SD, André BHM, Clerici MTPS (2021). Gluten-free rice & bean biscuit: characterization of a new food product. Heliyon.

[ref-55] Zhao Q, Selomulya C, Xiong H, Chen XD, Li X, Wang S, Bai C, Peng H, Zhou Q, Sun W (2014). Rice dreg protein as an alternative to soy protein isolate: comparison of nutritional properties. International Journal of Food Properties.

